# Analysis of Differences in Flavor Precursors Between Fast and Slow Muscles of Turpan Black Sheep Based on Lipidomics and Proteomics

**DOI:** 10.3390/foods15172994

**Published:** 2026-08-26

**Authors:** Yanni Ma, Na Li, Jiemila Maimaitijiang, Qian Ma, Shijie Bi, Yana Liu, Yong Chen, Batuer Abulikemu

**Affiliations:** 1 College of Food Science and Pharmacy, Xinjiang Agricultural University, Urumqi 830052, China; 15628172037@163.com (Y.M.); lina201127@163.com (N.L.); jamila1101@163.com (J.M.); m18799756952@163.com (Q.M.); bsj19941001@163.com (S.B.); lyn308@xjau.edu.cn (Y.L.); 2 College of Animal Science, Xinjiang Agricultural University, Urumqi 830052, China; xjaucy@163.com

**Keywords:** Turpan black sheep, muscle fiber type, flavor precursor, lipidomics, proteomics, integrated analysis

## Abstract

The skeletal muscle fiber type is a key determinant of meat quality. However, the molecular mechanisms underlying the differences in flavor precursors between fast-twitch and slow-twitch muscles in mutton remain unclear. The gluteus medius (GM, glycolytic type) and semitendinosus (ST, oxidative type) muscles of Turpan black sheep are two representative skeletal muscles that differ markedly in muscle fiber types. In this study, amino acids, fatty acids, lipidomics, and proteomics were integrated to systematically compare the molecular differences between the GM and ST. The results showed that the ST muscle had significantly higher levels of umami amino acids (glutamic acid, Glu; aspartic acid, Asp) and the mutton-specific flavor contributor gondoic acid (C20:1n-9) than the GM, whereas the GM exhibited higher contents of sweet-tasting amino acids (alanine, Ala; proline, Pro). Lipidomics and proteomics analyses identified 389 differentially abundant lipids and 480 differentially expressed proteins. Combined analysis indicated that the differential molecules were mainly enriched in glycerophospholipid metabolism and pathways regulating lipolysis, which are the main metabolic networks influencing the accumulation of flavor precursors. In addition, phosphatidylethanolamine (PE), phosphatidylcholine (PC), cardiolipin (CL), C20:1n-9, hexokinase 2 (HK2), pyruvate kinase M (PKM), myosin heavy chain 7 (MYH7), and myosin heavy chain 2 (MYH2) were identified as the key candidate markers. Overall, these data provide new insights into the molecular mechanisms by which muscle fiber type affects mutton flavor quality and offer potential molecular targets for improving mutton flavor through genetic selection and nutritional regulation.

## 1. Introduction

The flavor quality of animal-derived foods is a key factor in determining consumer acceptance and economic value [1]. Against the backdrop of continued global growth in meat consumption, consumers are placing increasing demands on the sensory quality of meat products, with flavor becoming one of the core attributes influencing purchasing decisions. The overall perception of flavor arises from the multi-layered interactions between volatile aroma compounds and non-volatile taste substances [2]. During thermal processing, various precursor substances present in meat matrices undergo chemical reactions, such as lipid oxidation, the Maillard reaction, and Strecker degradation, resulting in the formation of numerous volatile flavor compounds that collectively shape the characteristic flavor profile of meat products. Among these, free fatty acids are the principal precursors of heat-induced aroma compounds (such as aldehydes, alcohols, and ketones) [3], while free amino acids not only directly contribute to basic tastes such as umami and sweetness but also serve as substrates for the Maillard reaction and Strecker degradation, generating volatile flavor substances such as aldehydes and pyrazines upon heating [4]. Therefore, elucidating the differences in content and formation mechanisms of flavor precursors such as fatty acids and amino acids in muscle tissue is of great significance for understanding the upstream regulation of meat flavor quality.

Among livestock species, sheep meat stands out for its pronounced regional variation in consumer preferences, making it an ideal case for investigating the relationship between muscle biochemistry and flavor quality [5]. Sheep meat holds divergent cultural and economic significance across global markets. In China, sheep meat consumption is deeply embedded in food culture, particularly in northern and western regions, and is increasingly driven by the expansion of hot pot and barbecue restaurant chains [6]. Mediterranean consumers favor milder lamb [7], while Middle Eastern traditions prefer mutton with richer flavors in festive cuisine. Meanwhile, in Western markets such as Australia and Europe, consumer preference has shifted toward tender, mild-flavored lamb, with tenderness widely regarded as the primary determinant of eating quality [8]. These regional disparities highlight the importance of understanding how intrinsic muscle characteristics may influence consumer acceptance in different culinary contexts.

The skeletal muscle fiber type is an intrinsic determinant of muscle metabolic characteristics and biochemical composition [9]. Based on differences in myosin heavy chain (MyHC) isoforms, skeletal muscle fibers are generally classified into four types: slow oxidative (MyHC I), fast oxidative–glycolytic (MyHC IIa), fast glycolytic (MyHC IIb), and intermediate (MyHC IIx) fibers [10]. According to their metabolic features, these four types can be grouped into two major categories: fast-twitch fibers and slow-twitch fibers. It is well established that mammalian skeletal muscles are composed of a mixture of slow-twitch (type I) and fast-twitch (type II) fibers, and the fiber-type composition varies among different muscles depending on their anatomical location and functional demands. In sheep, multiple studies have demonstrated that glycolytic fast-twitch fibers (type II) predominate in most skeletal muscles [11], with MyHC IIx being the predominant fast glycolytic fiber type. The gluteus medius (GM) and semitendinosus (ST) muscles used in this study are two distinct skeletal muscles with markedly different fiber-type proportions and metabolic characteristics. The GM is a glycolytic-type muscle dominated by fast-twitch fibers, whereas the ST in Turpan black sheep is an oxidative-type muscle enriched in slow-twitch fibers. Fast-twitch fibers rely predominantly on glycolysis for energy supply and are rich in type II myosin heavy chains, whereas slow-twitch fibers are enriched in mitochondria and myoglobin and mainly utilize oxidative phosphorylation as their metabolic pathway [12]. This metabolic divergence determines the fundamental differences in the lipid and protein compositions of different muscle fiber types. Slow-twitch fibers, owing to their active oxidative metabolism, contain higher levels of phospholipids and mitochondrial membrane lipids, whereas fast-twitch fibers are characterized by abundant glycogen and glycolysis-related enzyme systems [13]. Consistent with these metabolic differences, the GM exhibits higher shear force and lower water-holding capacity, whereas the ST displays superior tenderness, water-holding capacity, and a more favorable lipid and amino acid profile. These inherent differences in quality traits are closely linked to their respective metabolic characteristics and determine their suitability for different culinary applications. Because of these pronounced differences in lipid and protein composition, fast- and slow-twitch fibers constitute an ideal natural model for investigating the intrinsic differences in muscle flavor precursors [14]. However, the molecular regulatory mechanisms underlying the differences in flavor precursors between fast- and slow-twitch muscles in lambs remain to be systematically elucidated.

Conventional approaches to evaluating muscle flavor precursors rely on measuring fatty acid and amino acid contents [15]. While useful for describing phenotypic differences, such measurements cannot reveal the molecular mechanisms that govern precursor accumulation. Recent advances in lipidomics and proteomics have provided powerful tools to overcome this limitation. Lipids are key determinants of meat flavor, influencing nutritional quality, the formation of volatile compounds, sensory properties, and other meat quality traits [16]. Among these, the role of lipids in flavor development is particularly critical, as they shape overall sensory characteristics and directly affect consumer acceptance [17]. Previous studies have compared the mitochondrial lipid profiles of the longissimus dorsi and psoas major muscles in cattle, and more recently, untargeted lipidomics has been used to explore how flaxseed oil-derived cyclopeptides modulate lipid degradation in high-fat beef during storage [18]. Likewise, proteomics enables systematic characterization of key enzymes involved in lipid hydrolysis and protein degradation [19]. Although both omics technologies have been widely applied in meat science, no study has yet integrated them in lamb muscles to systematically dissect the flavor-precursor differences between fast- and slow-twitch lamb muscles. This underscores the need for an integrated multi-omics strategy to comprehensively elucidate, from both compositional and functional regulation perspectives, the molecular basis of flavor precursor differences in these two muscle types.

In this study, the GM (glycolytic) and ST (oxidative) muscles of Turpan black sheep were used as experimental models. After systematically comparing their amino acid and fatty acid profiles, we integrated lipidomics and proteomics to characterize the molecular basis of flavor precursor differences between fast- and slow-twitch muscles. Differential lipids were identified by partial least squares discriminant analysis (PLS-DA), and key regulatory molecules were uncovered through combined protein–lipid correlation analysis. The results are expected to inform strategies—including genetic selection, nutritional manipulation, and production management—for improving lamb meat flavor quality.

## 2. Materials and Methods

### 2.1. Sample Preparation

Six purebred Turpan black rams, approximately 12 ± 1.5 months of age and with an average body weight of 42.50 ± 2.30 kg, were used in this study. All experimental animals were sourced from Fengcheng State-owned Pasture Co., Ltd. (Tuokexun County, Turpan City, Xinjiang, China) and were raised under the same grazing and supplemental feeding regimes. The basal diet consisted mainly of native pasture forages, supplemented with appropriate amounts of alfalfa, clover, and hay. Concentrate supplementation was formulated to meet the nutritional requirements of growing lambs, with energy and protein levels appropriately adjusted. The sheep were fasted for 12 h before slaughter with free access to water. Slaughter procedures strictly followed the Chinese national standard. Within 30 min postmortem, samples of the GM and ST muscles were collected from the right hind limb of each carcass. After removing visible connective tissue and fat, considering that skeletal muscle is composed of a mixture of slow-twitch and fast-twitch fibers and that the proportion of fiber types may vary across different regions of the same muscle, a mixed sampling strategy was adopted in this study to minimize sampling bias. Specifically, the entire muscle was completely homogenized to further reduce intramuscular variability. The resulting homogenate was used as the representative analytical sample for all subsequent determinations. The homogenized samples were immediately snap-frozen in liquid nitrogen and stored at −80 °C for subsequent analyses of hydrolyzed amino acids, fatty acids, lipidomics, and proteomics.

### 2.2. Hydrolyzed Amino Acids

Hydrolyzed amino acids were quantified by reverse-phase high-performance liquid chromatography (HPLC) following Qi et al. [20]. Briefly, 1.0 g of muscle was hydrolyzed with 10 mL of 6 mol/L HCl at 110 °C for 24 h. The hydrolysate was dried under reduced pressure at 45 °C, reconstituted with 12 mL of 0.1 mol/L HCl, and adjusted to 20 mL with ultrapure water. For phenyl isothiocyanate (PITC) derivatization, 200 μL of the sample was mixed with 20 μL of norleucine (internal standard, 0.02 mol/L), 100 μL of triethylamine–acetonitrile (1 mol/L), and 100 μL of PITC–acetonitrile (0.2 mol/L), reacted for 1 h at room temperature, and extracted with 400 μL of n-hexane. The lower layer was diluted 1:4 (*v*/*v*) with ultrapure water and filtered (0.22 μm). HPLC was performed on a Shimadzu system (Shimadzu, Kyoto, Japan) with a Diamonsil AAA column (250 mm × 4.6 mm, Dikma, Beijing, China) using a gradient of sodium acetate buffer (pH 6.50) and methanol–acetonitrile–water (20:60:20) at 1.0 mL/min and 35 °C, with detection at 254 nm. Results were expressed as mg/g fresh tissue. To evaluate the sensory relevance of individual amino acids, the taste activity value (TAV) of each amino acid was calculated as the ratio of its concentration to its taste threshold. The TAV was calculated using the following formula:

$$\mathrm{TAV}=\frac{C_{i}}{T_{i}}$$

where C_i_ is the concentration of amino acid i (mg/g fresh tissue), and T_i_ is the taste thresh old of amino acid i (mg/g).

### 2.3. Fatty Acids

Briefly, 50 mg of muscle was homogenized with 1 mL of dichloromethane–methanol (1:1, *v*/*v*) at 50 Hz for 3 min in a refrigerated tissue grinder. The mixture was ultrasonicated at low temperature for 15 min, stood at −20 °C for 15 min, and centrifuged at 13,000× *g* for 10 min at 4 °C. The supernatant (500 μL) was evaporated to dryness under nitrogen. The residue was methylated with 0.5 mL of 0.2 mol/L KOH in methanol at 60 °C for 30 min. After cooling, 0.5 mL of n-hexane was added, and the mixture was vortexed and centrifuged at 13,000× *g* for 10 min at 4 °C. The upper phase (100 μL) was transferred and analyzed by gas chromatography–mass spectrometry (GC–MS) on an Agilent 8890-7000D system (Agilent Technologies, Santa Clara, CA, USA) with a DB-FAST FAME capillary column (30 m × 250 μm × 0.25 μm; Agilent J&W Scientific, Folsom, CA, USA). Helium was the carrier gas at 1.0 mL/min. The injector was at 250 °C, with a 1 μL injection (split 10:1) and a solvent delay of 1.85 min. The GC column temperature was programmed to hold at 80 °C for 30 s and rise to 175 °C at a rate of 70 °C per minute, then rise to 230 °C at a rate of 8 °C per minute, finally hold at temperature of 230 °C for 2 min. Mass spectra were acquired in electron impact (EI) mode at 70 eV, with ion source, quadrupole, and transfer line temperatures of 250, 150, and 240 °C, respectively, in selected ion monitoring (SIM) mode which was chosen to enhance sensitivity and selectivity for the targeted quantification of fatty acid methyl esters. Fatty acids were identified by comparison with a 36-component FAME standard (CDAA-252795, ANPEL) and quantified by the external standard method without the use of an internal standard. Results were expressed as mg/100 g muscle tissue.

### 2.4. Lipidomics

Lipids were extracted using the MTBE method. A 50 mg muscle sample was placed in a 2 mL tube with a 6 mm grinding bead, 280 µL extraction solvent (methanol:water = 2:5, *v*/*v*), and 400 µL methyl tert-butyl ether (MTBE). The mixture was homogenized in a refrigerated tissue grinder for 6 min (−10 °C, 50 Hz), ultrasonicated for 30 min (5 °C, 40 kHz), and kept at −20 °C for 30 min. After centrifugation at 13,000× *g* for 15 min at 4 °C, 350 µL of the upper (organic) phase was dried under nitrogen. The residue was reconstituted in 100 µL of isopropanol: acetonitrile (1:1, *v*/*v*), vortexed, ultrasonicated in an ice-water bath for 5 min, and centrifuged at 13,000× *g* for 10 min at 4 °C. The final supernatant was transferred to an autosampler vial for LC-MS analysis. Pooled QC samples were prepared by combining equal volumes of all sample extracts. QC samples were injected after every 10 sample injections throughout the analytical run to monitor system stability and reproducibility. A total of six biological replicates were analyzed for each muscle group.

Chromatographic separation was performed on a Thermo Fisher Ultimate 3000 UHPLC system (Thermo Fisher Scientific, Waltham, MA, USA) equipped with an Accucore C30 column (100 mm × 2.1 mm × 2.6 µm). Mobile phase A was acetonitrile–water = 50:50 (*v*/*v*) (containing 0.1% formic acid and 10 mmol/L ammonium acetate), and mobile phase B was acetonitrile: isopropanol: water (10:88:2, *v*/*v*/*v*, containing 0.02% formic acid and 2 mmol/L ammonium acetate). The flow rate was 0.40 mL/min, injection volume was 3 µL, and column temperature was 40 °C. The gradient was: 0–3 min, B 35% to 60%; 3–10 min, B 60% to 85%; 10–12.5 min, B 85% to 100%; held at 100% until 13.9 min; then returned to 35% at 14 min and held until 16 min.

Mass spectrometry was performed on a Q-Exactive HF-X mass spectrometer (Thermo Fisher Scientific, Waltham, MA, USA) in both positive and negative ion modes (*m*/*z* 200–2000). The spray voltage was +3000 V (positive) and −3000 V (negative), with sheath gas at 60 arb, auxiliary gas at 20 arb, auxiliary gas heater temperature of 370 °C, HESI source capillary temperature of 320 °C, and stepped collision energies of 20–40–60 V. Full MS scans were performed at a resolution of 120,000 (at *m*/*z* 200) with an AGC target of 1 × 10^6^ and a maximum injection time of 50 ms. For each full scan, the top 10 most intense precursor ions were selected for MS/MS fragmentation. MS/MS spectra were acquired at a resolution of 30,000 with an AGC target of 1 × 10^5^, a maximum injection time of 50 ms, a precursor isolation window of 1.5 *m*/*z*, and an S-Lens RF level of 45. Data were acquired in data-dependent acquisition (DDA) mode.

### 2.5. Proteomics

Proteomic analysis of the GM and ST muscles was performed using a Vanquish Neo UHPLC system (Thermo Fisher Scientific, USA) coupled to an Orbitrap Astral mass spectrometer (Thermo Fisher Scientific). 50 mg of muscle sample was added to 500 µL of lysis buffer containing protease inhibitors (8 mol/L urea, 1% SDS), and homogenized in a refrigerated tissue grinder at −10 °C and 50 Hz for 3 cycles of 180 s each, followed by low-temperature ultrasonication for 30 min. After centrifugation, the supernatant was collected, and the protein concentration was determined using the BCA assay. An aliquot of 100 μg protein was mixed with 100 mmol/L TEAB and 10 mmol/L TCEP and incubated at 37 °C for 60 min, followed by the addition of 40 mmol/L iodoacetamide and incubation in darkness for 40 min. Proteins were precipitated with acetone and centrifuged, and the resulting pellet was digested with trypsin at an enzyme-to-protein ratio of 1:50 (*w*/*w*) at 37 °C for 8 h. The resulting peptides were then desalted using a Waters Oasis HLB solid-phase extraction cartridge (30 mg, Waters, Milford, MA, USA). The cartridge was sequentially activated with 1 mL methanol and equilibrated with 1 mL 0.1% TFA. After loading the peptide sample, the cartridge was washed with 1 mL 0.1% TFA to remove salts, and the peptides were eluted with 1 mL 80% acetonitrile/0.1% TFA. The eluate was then dried in a vacuum concentrator and quantified.

For LC-MS/MS analysis, peptide separation was performed on a Vanquish Neo system (Thermo Fisher Scientific, Waltham, MA, USA) using an in-house packed column (15 cm × 100 μm, 1.7 μm). Mobile phase A consisted of 2% acetonitrile and 0.1% formic acid, and mobile phase B consisted of 80% acetonitrile and 0.1% formic acid. The flow rate was 1 μL/min with gradient elution. Peptides were analyzed using an Astral mass spectrometer (Thermo Fisher Scientific, Waltham, MA, USA) operated in DIA mode with a spray voltage of 2.2 kV. The MS1 and MS2 scan ranges were set to *m*/*z* 380–980 and *m*/*z* 150–2000, respectively. Raw data were processed using Spectronaut TM 19 software with a library-free approach (direct DIA) and searched against the NCBI (Ovis aries) database allowing a maximum of two missed cleavages with a false discovery rate (FDR) of 1%.

### 2.6. Statistical Analysis

For amino acid and fatty acid data: All statistical analyses were performed using R software (version 4.3.2, R Core Team). Differences in amino acid and fatty acid concentrations between GM and ST muscles were compared using the independent samples *t*-test, with the significance level set at α = 0.05. The experimental unit was the individual animal (n = 6 per muscle group), with each muscle sample representing one biological replicate. Results are presented as mean ± standard deviation (SD) to describe the variability among individual samples within each group. In addition, the standard error of the mean (SEM) is provided in the tables as a measure of the precision of the estimated mean.

For untargeted lipidomics data: All statistical analyses were performed with the individual muscle as the experimental unit (six biological replicates per group for GM and ST), using R software (version 4.3.2, with the ropls package version 1.6.2) and Python (version 3.9, with SciPy version 1.12.0). To improve data normality and stabilize variance, the normalized lipidomics response intensity data were subjected to log_10_ transformation. The transformed data were used for subsequent statistical analyses, but intergroup differences were presented as fold changes (FC) and direction of change on the original scale. For multivariate analysis, principal component analysis (PCA) and orthogonal partial least-squares discriminant analysis (OPLS-DA) were performed with the ropls package, and model stability was evaluated through 7-fold cross-validation. For univariate analysis, an independent samples *t*-test was used to compare differences between the GM and ST groups, with the significance level set at α = 0.05. Differential lipids were selected based on variable importance in projection (VIP) values from the OPLS-DA model (VIP > 1.0) and Student’s *t*-test *p*-values (*p* < 0.05). The identified differential lipids were annotated against the KEGG database, and pathway enrichment analysis was conducted using Fisher’s exact test, with *p* < 0.05 considered the threshold for significant enrichment.

For proteomics data: Proteomic quantitative data were identified and analyzed using Spectronaut^TM^ 19 software with the MaxLFQ algorithm for protein quantification. The independent samples *t*-test in R software was employed to compare the significant differences between the two groups (GM vs. ST). Proteins with *p* < 0.05 and an absolute fold change (FC) > 1.2 were considered differentially expressed proteins. Prior to statistical testing, he data were log_2_-transformed to stabilize variance. The experimental unit was a single animal individual, with six biological replicates per group, and the significance level was set at α = 0.05. The identified differentially expressed proteins were annotated using the GO database (http://geneontology.org/) from three aspects: biological process, cellular component, and molecular function. Additionally, metabolic pathway enrichment analysis was performed using the KEGG database (http://www.genome.jp/kegg/ (accessed on 24 August 2026)).

For correlation analysis: Pearson correlation coefficients were used to assess the associations between differential lipids and proteins, with |r| > 0.5 and *p* < 0.05 considered statistically significant. The correlation coefficient reflects the direction and strength of association, while statistical significance was determined by *p*-values compared to the preset α = 0.05 level.

## 3. Results

### 3.1. Amino Acid Analysis

The amino acid contents of the GM and ST muscles are presented in Table 1. The total amino acid (TAA) content was significantly higher in ST than in GM (*p* = 0.001).

Regarding individual amino acids, the contents of aspartic acid (Asp), glutamic acid (Glu), serine (Ser), arginine (Arg), tyrosine (Tyr), valine (Val), methionine (Met), isoleucine (Ile), and leucine (Leu) were significantly higher in ST than in GM (*p* < 0.05), whereas the levels of histidine (His), threonine (Thr), alanine (Ala), and proline (Pro) were significantly higher in GM than in ST (*p* < 0.05).

In terms of taste activity, Glu exhibited the highest TAV in both muscles (64.35 in GM and 75.39 in ST), indicating that umami was the predominant taste attribute, with a more pronounced umami taste in ST. Among sweet-tasting amino acids, the TAVs of Ala, Thr, and Pro were markedly higher in GM than in ST, consistent with their significantly higher concentrations in GM, suggesting a sweeter taste profile for GM. For bitter-tasting amino acids, the TAVs of Met, Val, Ile, Leu, Arg, and Lys were higher in ST than in GM, whereas His showed a higher TAV in GM, consistent with its higher concentration in GM.

### 3.2. Fatty Acid Analysis

As shown in Table 2, the contents of caproic acid (C6:0), undecanoic acid (C11:0), tridecanoic acid (C13:0), and eicosenoic acid (C20:1n-9) were significantly higher in ST than in GM (*p* < 0.05), whereas the contents of the other fatty acids did not differ significantly between ST and GM (*p* > 0.05). The fatty acids (SFA), monounsaturated fatty acids (MUFA), and polyunsaturated fatty acids (PUFA) contents were higher in ST than in GM, but the differences were not significant. The PUFA/SFA ratios of the GM and ST were 0.244 and 0.243, respectively.

### 3.3. Lipidomics Analysis

**Figure 1:**
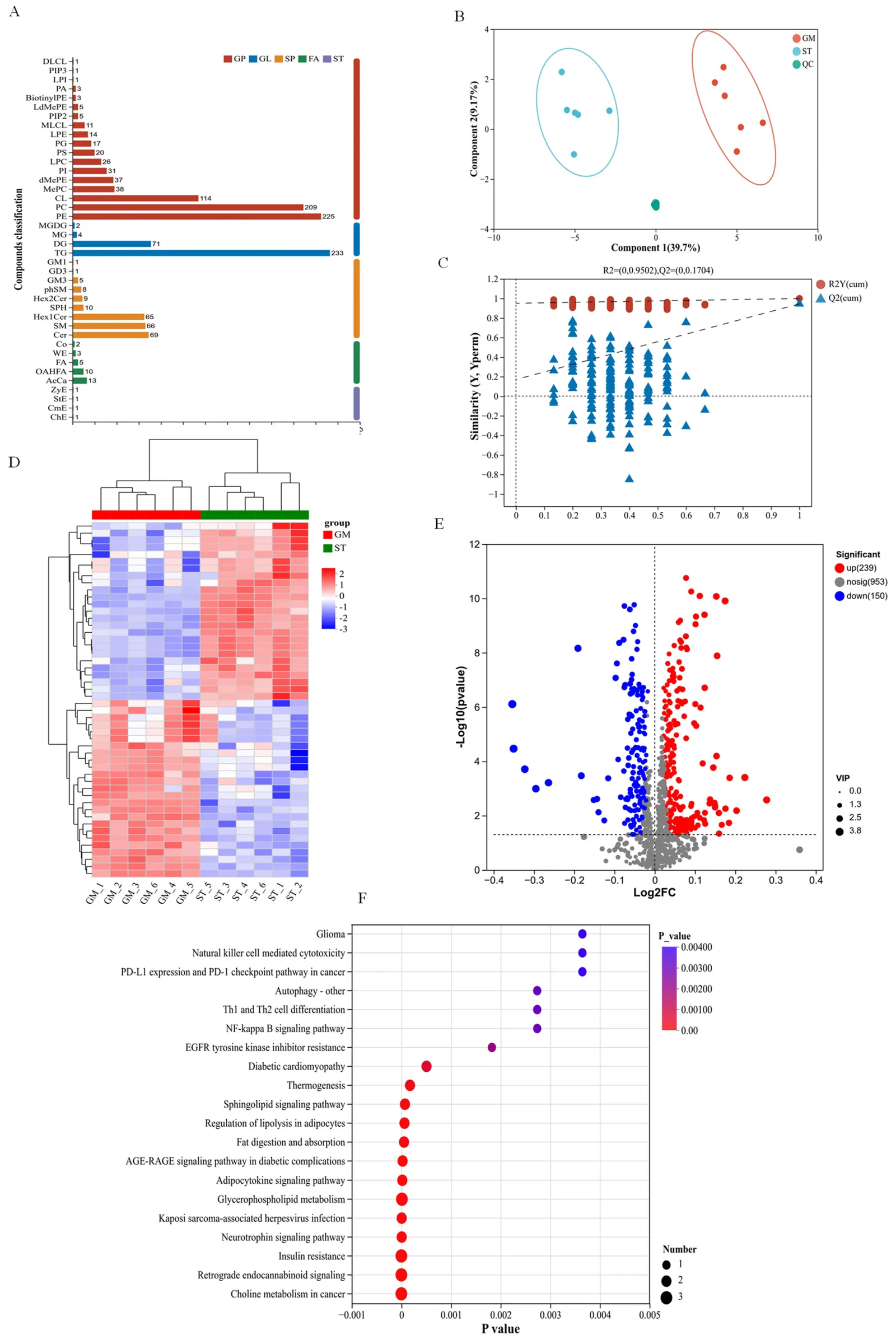
LC-MS-based lipidomic profiling of muscle tissues. (**A**) Lipid subclass composition and molecular species counts; (**B**) PLS-DA score plot; (**C**) permutation test; (**D**) volcano plot of DALs; (**E**) hierarchical clustering heatmap of DALs; (**F**) KEGG pathway enrichment analysis of DALs (top 20 pathways).

In this study, we analyzed the lipid profiles of GM and ST muscles. Using LC-MS/MS, a total of 1342 lipid species were identified in the muscle samples (Figure 1A), which were assigned to 40 lipid subclasses. In terms of molecular species number, most lipids were classified as triacylglycerols (TG; 233, 17.36%), followed by phosphatidylethanolamines (PE; 225, 16.77%) and phosphatidylcholines (PC; 209, 15.57%).

PLS-DA was used to analyze the differences in lipid profiles between the GM and ST muscle samples. The PLS-DA results showed a clear separation of the lipidomic profiles between the two groups (Figure 1B), with model parameters R^2^Y 0.986 and Q^2^ 0.879, suggesting good model fit and predictive performance. A permutation test was performed to verify whether the model was overfitted (n = 200, Figure 1C). The results showed a negative slope of the Q^2^ regression line, with a Q^2^ intercept of 0.170 and an R^2^ intercept of 0.950, indicating that the model was not overfitted.

Differentially abundant lipids (DALs) between GM and ST muscles were screened using *p* < 0.05 and VIP > 1 as the criteria. A total of 389 DALs were identified of which 239 were upregulated and 150 were downregulated in the GM (Figure 1E). These DALs were assigned to 28 lipid subclasses. Based on the number of molecular species, the top five subclasses of DALs were triacylglycerols (TG), phosphatidylethanolamines (PE), phosphatidylcholines (PC), cardiolipin (CL), and hexosylceramides (Hex1Cer). Among them, the differential molecular species of PC and Hex1Cer were predominantly upregulated. Hierarchical clustering analysis of DALs (Figure 1D) showed clear clustering of the six biological replicates of GM and ST muscles, indicating distinct patterns of differential lipid compositions between the two muscle types.

To further elucidate the biological functions associated with the DALs, KEGG pathway enrichment analysis was performed on the 389 DALs. A total of 90 pathways were significantly enriched (*p* < 0.05). According to the enrichment significance (Figure 1F), the most significantly enriched pathway was Choline metabolism in cancer, followed by Insulin resistance and Retrograde endocannabinoid signaling. Among the pathways related to lipid metabolism, Glycerophospholipid metabolism was the most significantly enriched, followed by Fat digestion and absorption and the Adipocytokine signaling pathway. The enriched pathways were mainly involved in three major functional categories: Metabolism, Environmental Information Processing, and Organismal Systems.

### 3.4. Proteomics Analysis

**Figure 2:**
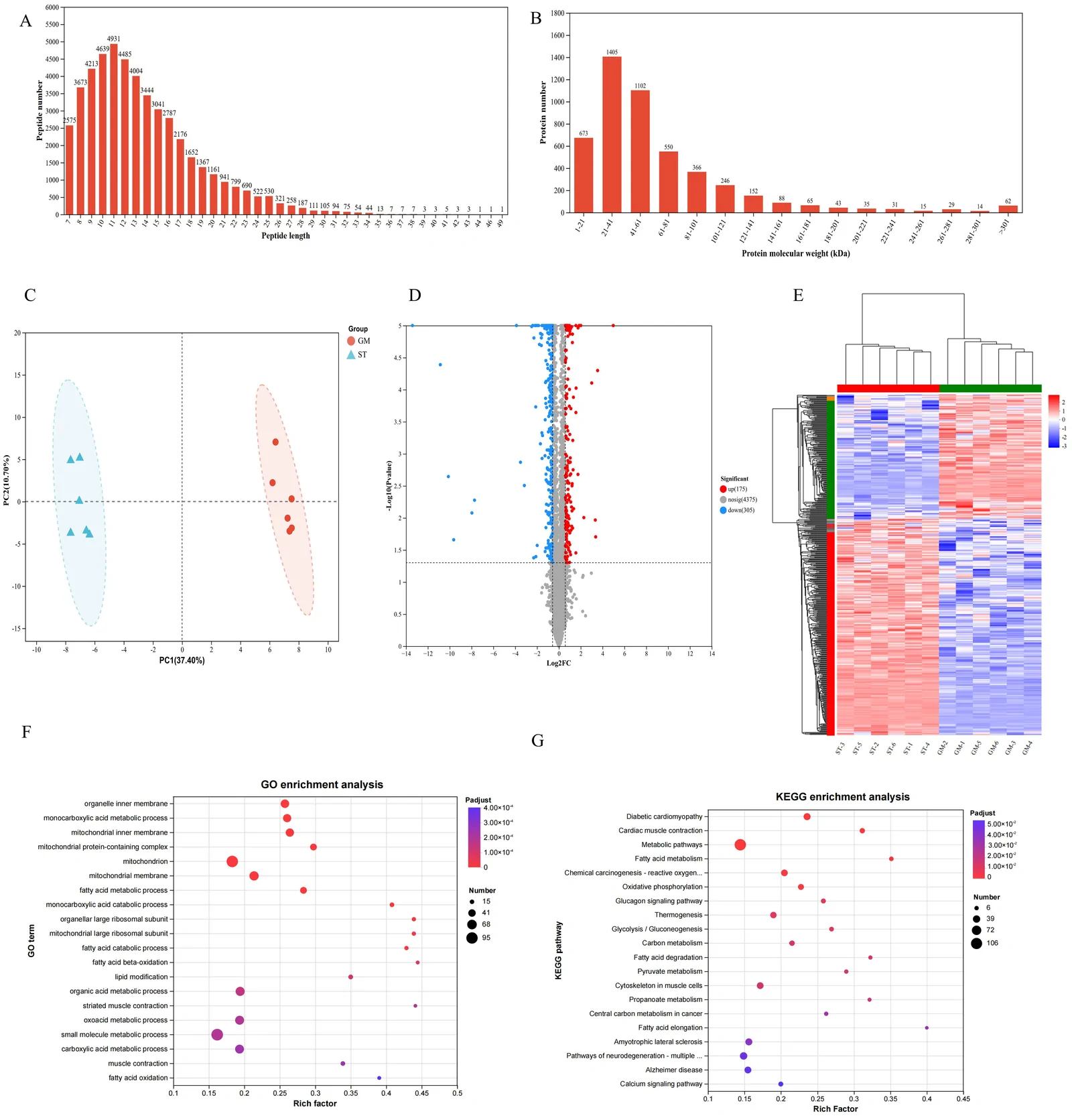
Bioinformatics analysis of proteomic data. (**A**) Peptide length distribution; (**B**) molecular weight distribution; (**C**) PCA score plot; (**D**) volcano plot of DEPs; (**E**) hierarchical clustering heatmap of DEPs; (**F**) GO functional enrichment analysis (top 20 GO terms); (**G**) KEGG pathway enrichment analysis (top 20 pathways).

Using DIA-based quantitative proteomics, we identified 48,933 peptides and 4876 proteins in the GM and ST muscles. Among these peptides, 90.22% consisted of 7–20 amino acids (Figure 2A), and 62.69% of the identified proteins had molecular weights between 20 and 80 kDa (Figure 2B), confirming the reliability of the proteomic data. Principal component analysis (PCA) showed that PC1 and PC2 explained 37.40% and 10.70% of the variance, respectively, with a cumulative contribution of 48.10% (Figure 2C). The GM and ST muscles were clearly separated in the PCA score plot, and the GM were more tightly clustered, suggesting good biological reproducibility.

To screen for differentially expressed proteins (DEPs) associated with muscle fiber type, differential expression analysis was performed between GM and ST. Compared with ST, a total of 480 DEPs were identified in GM, of which 175 were upregulated and 305 were downregulated (Figure 2D). Among them, the slow-twitch fiber marker MYH7 was significantly upregulated in ST, whereas the fast-twitch fiber marker MYH2 was significantly upregulated in GM; fast skeletal troponin T3 (TNNT3) was also significantly upregulated in GM. In addition, two key glycolytic enzymes, hexokinase-2 (HK2) and pyruvate kinase (PKM), were significantly upregulated in GM, indicating a stronger glycolytic capacity in fast-twitch muscle. Cluster analysis showed that the protein expression profiles of GM and ST samples were clearly divided into two major clusters corresponding to the two muscle types (Figure 2E), suggesting that the DEPs are closely related to the distinct metabolic phenotypes of different muscle fiber types.

To further explore the biological functions of DEPs, GO and KEGG pathway enrichment analyses were performed. GO enrichment analysis (Figure 2F) showed that the top 20 significantly enriched GO terms were mainly associated with Biological Process (BP) and Cellular Component (CC). In the BP category, in addition to small-molecule metabolic process (GO:0044281) and organic acid metabolic process (GO:0006082), terms such as monocarboxylic acid metabolic process, fatty acid metabolic process, fatty acid catabolic process, fatty acid β-oxidation, lipid modification, and muscle contraction were significantly enriched. In the CC category, DEPs were primarily enriched in mitochondrion, mitochondrial inner membrane, organelle inner membrane, mitochondrial protein complex, and mitochondrial large ribosomal subunit. These results suggest that DEPs may primarily affect mitochondrial function and lipid metabolism-related processes. KEGG pathway enrichment analysis (Figure 2G) further revealed that DEPs were significantly enriched in pathways including fatty acid metabolism, oxidative phosphorylation, cardiac muscle contraction, carbon metabolism, muscle cell cytoskeleton, glycolysis/gluconeogenesis, thermogenesis, glucagon signaling pathway, fatty acid degradation, and pyruvate metabolism. Among these, fatty acid metabolism showed the highest enrichment factor and the smallest *p* value, indicating the most significant enrichment; oxidative phosphorylation, cardiac muscle contraction, and carbon metabolism also exhibited high significance. These pathways are primarily involved in energy metabolism, lipid metabolism, and muscle contraction, suggesting that the proteomic differences between GM and ST may regulate muscle phenotypes by modulating these key pathways.

### 3.5. Integrative Analysis of Lipidomics and Proteomics

To elucidate the relationship between lipid metabolites and proteins, correlation analysis was performed between 389 DALs and 480 DEPs. The results revealed extensive correlations between lipid metabolites and proteins (|r| > 0.5), with positive correlations mainly observed between PE/PC and slow-twitch muscle–related proteins, whereas negative correlations were primarily found between SM and fast-twitch muscle–related proteins.

**Figure 3:**
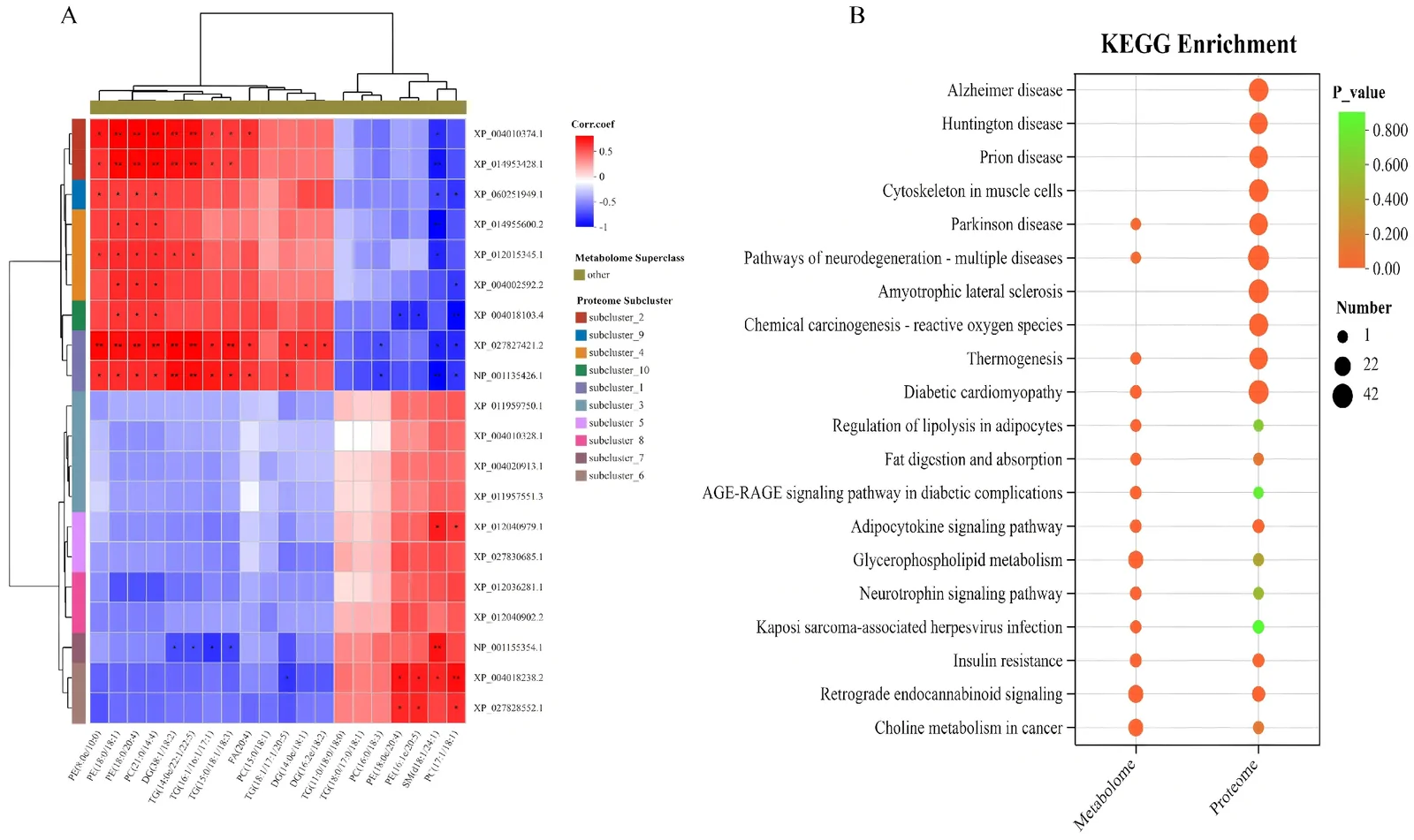
Integrative lipidomics and proteomics analysis. (**A**) Hierarchical clustering heatmap of DALs and DEPs (*, *p* < 0.05; **, *p* < 0.01); (**B**) KEGG pathway enrichment analysis of DALs and DEPs (top 20 pathways).

As shown in Figure 3A, the slow-twitch muscle fiber marker protein CA3 was strongly positively correlated with PE (18:0/20:4), PE (18:0/18:1), and PC (21:0/14:4) (r = 0.741, *p* = 0.006), but strongly negatively correlated with SM (d18:1/24:1) (r = −0.748, *p* = 0.005). This suggests that the slow oxidative metabolic phenotype represented by CA3 is aligned with phospholipid accumulation but opposite to that of sphingomyelins. In addition, the key glycolytic enzyme ENO3 was positively correlated with SM (d18:1/24:1) (r = 0.685, *p* = 0.014), further supporting the notion that fast glycolytic muscle and slow oxidative muscle differ fundamentally in their lipid composition.

Furthermore, a joint KEGG enrichment analysis of the DALs and DEPs (Figure 3B) identified multiple pathways related to lipid metabolism, energy metabolism, and muscle contraction. Among these, the glycerophospholipid metabolism pathway showed the highest enrichment and represented the most distinct functional module between the two muscle types. The pathway regulating lipolysis in adipocytes also showed a clear enrichment trend, indicating divergent lipolytic activity between GM and ST muscles. Additionally, the enrichment of the muscle cell cytoskeleton pathway reflected the differences in structural protein composition and contractile properties between the two muscles. Notably, several pathways related to mitochondrial function and energy homeostasis (such as thermogenesis and insulin resistance) were also enriched to varying degrees, suggesting intrinsic differences in oxidative metabolic capacity between the two muscles. Collectively, these findings indicate that the molecular differences between GM and ST muscles mainly focus on lipid metabolism and energy utilization, providing a metabolic basis for understanding the mechanisms of flavor precursor accumulation in a muscle fiber type–dependent manner.

## 4. Discussion

Skeletal muscle fiber type is a key determinant of flavor quality in lamb meat. Among these fiber types, oxidative fibers, which are rich in lipids and flavor-related proteins, generally exhibit superior flavor characteristics compared to glycolytic fibers, making different muscle fiber types an ideal model for dissecting flavor differences in meat [21]. Although previous studies on different anatomical muscles of pigs and cattle have demonstrated the widespread impact of muscle fiber type on meat quality [20,22], the specific flavor contributions of fast-twitch (type II) and slow-twitch (type I) fibers in different lamb cuts remain unclear, particularly regarding their lipidomic and proteomic profiles.

To address this gap, we first compared the amino acid and fatty acid profiles of GM and ST muscles. The amino acid composition directly influences the taste quality of meat [23]. However, statistical significance does not necessarily equate to sensory perceptibility. To assess the potential sensory relevance of the observed differences in amino acid concentrations between GM and ST muscles, we calculated the taste activity values (TAV) of individual amino acids by dividing their concentrations by their respective taste thresholds reported in the literature. The TAV analysis revealed that umami was the predominant taste attribute in both muscles, with a more pronounced umami character in ST. Among the sweet-tasting amino acids, the TAVs of Ala, Thr, and Pro were markedly higher in GM than in ST, suggesting that GM may possess a sweeter taste profile. Based on these TAV differences, we hypothesize that ST may be characterized by a more umami-dominant taste, whereas GM may tend toward a sweetness-dominant profile. However, this hypothesis requires validation through sensory evaluation using trained taste panels and consumer acceptability testing, as the combined effects of multiple taste-active compounds cannot be predicted from individual TAVs alone. Oxidative fibers possess a higher oxidative metabolic capacity, which promotes the accumulation of flavor-related amino acids, such as glutamate [24]. In contrast, the enrichment of Ala and Pro in glycolytic fibers may be associated with intermediates of their active glycolytic metabolism [25].

In terms of fatty acid composition, the level of eicosenoic acid (C20:1n-9) was significantly higher in ST than in GM. In addition, although the oleic acid and linoleic acid contents in ST did not differ significantly from those in GM, their oxidative degradation products (aldehydes, alcohols, and ketones) directly participate in aroma formation during thermal processing, indicating that the ST may have a greater potential for cooked flavor development. Although the present study did not conduct volatile compound analysis on cooked meat, the fatty acid composition data allow for a literature-based inference regarding the potential for volatile flavor formation. Unsaturated fatty acids, particularly C18:1n-9 and C18:2n-6, are well-documented precursors of volatile aldehydes, alcohols, and ketones via thermal oxidation during cooking. In this study, the ST muscle showed numerically higher contents of oleic acid and linoleic acid, suggesting that ST may have a greater substrate availability for lipid-derived aroma generation. The significantly higher level of C20:1n-9 in ST further supports this notion, as monounsaturated fatty acids with chain lengths ≥ C18 are known to contribute to the formation of characteristic meaty and fatty aroma notes upon thermal degradation [26]. Furthermore, the total PUFA content was higher in ST than in GM, which is particularly relevant because PUFAs are more susceptible to oxidative degradation than SFAs or MUFAs, thus serving as key precursors for the generation of short-chain aldehydes (e.g., hexanal, nonanal) that are critical to cooked meat aroma [27]. These findings suggest that the fatty acid composition of ST muscle, characterized by higher levels of both MUFAs and PUFAs, may confer a greater potential for the development of volatile flavor compounds upon heating, although direct experimental verification through GC-MS analysis is required to confirm this hypothesis. The differences in fatty acid composition may stem from the higher mitochondrial content and fatty acid metabolic activity in oxidative muscle fibers. Oxidative muscles are rich in mitochondria, whose membrane phospholipids contain a higher proportion of polyunsaturated fatty acids, while enhanced β-oxidation enzyme activity promotes fatty acid turnover and remodeling, thereby shaping a characteristic fatty acid profile [28].

The differences in fatty acid composition indicate that muscle fiber type may shape distinct fatty acid profiles by influencing lipid metabolism. However, fatty acid analysis only reflects differences in total fatty acid composition and cannot reveal the profiles of specific lipid molecular species. In recent years, lipidomics has provided powerful tools for elucidating the relationship between muscle lipid composition and flavor quality. A recent study using a targeted lipidomics approach identified 1080 lipid molecules belonging to 41 subclasses in Tan lamb meat from different regions [29]. In the present study, 1342 lipid molecules were detected, covering 40 lipid subclasses, and the number of identified species exceeded the 1080 previously reported in Tan lambs. This suggests that the ovine lipidome is more complex than previously appreciated, and our work contributes to a deeper understanding of the lipid molecular basis underlying flavor differences between slow- and fast-twitch muscles.

Lipidomic analysis showed that phosphatidylcholine (PC), triacylglycerol (TG), and phosphatidylethanolamine (PE) were the three predominant lipid subclasses in ovine muscle. From the perspective of flavor contribution, PC and PE are rich in polyunsaturated fatty acids and are thus prone to oxidative degradation during heating, serving as key precursors of volatile flavor compounds such as aldehydes and alcohols [30]. Liu et al. reported that specific PC and PE molecules are closely associated with the formation of major aroma compounds in roasted lamb [31]. In the present study, the PE level was significantly higher in the slow-twitch ST than in the fast-twitch GM, suggesting that the ST may possess more favorable lipid substrate conditions. PC (18:1_18:1) has been reported to be positively correlated with a variety of flavor compounds [32], whereas PE (18:1_18:1) shows a negative correlation with flavor compounds such as butyric acid [33], indicating that differences in the composition of specific phospholipid molecules may directly contribute to the differences in flavor characteristics between fast- and slow-twitch muscles. TG is the main storage form of intramuscular fat, and its fatty acid composition directly influences the profile of volatile flavor compounds generated during heating. Liu et al. found that TG (16:0_18:1_18:1) and TG (18:0_18:0_18:1) are the major lipid species associated with lamb aroma [34]. Zhou et al. demonstrated that TG (16:0_18:1_18:1), TG (16:0_18:1_18:2), and TG (16:0_16:1_18:1) play important roles in the formation of key aroma compounds in beef [35], suggesting that specific TG molecules modulate the release patterns of aroma precursors during thermal processing through their fatty acid composition. Cardiolipin (CL) is mainly located in the inner mitochondrial membrane and plays a critical role in maintaining the stability of the mitochondrial respiratory chain [36].

Lipidomic data revealed differential distributions of flavor precursor lipids between fast- and slow-twitch muscles. However, these findings only reflect static molecular differences and cannot elucidate the mechanisms governing their formation. Proteins are the direct executors of metabolic functions in muscles, and their differential expression determines the metabolic characteristics of different muscle fiber types, thereby influencing the accumulation of various flavor precursors [37]. In recent years, DIA-based quantitative proteomics has been widely applied to investigate the protein expression profiles associated with meat quality [38]. In the present study, 480 DEPs were identified. The expression patterns of these DEPs may explain the differences in flavor precursor lipids observed in the lipidomic analysis at the enzymatic level. The differential expression of marker proteins in muscle fiber types establishes the structural basis for flavor differences. MYH7 and MYH2 are marker proteins for slow-twitch and fast-twitch fibers, respectively [39]. In this study, MYH7 was significantly downregulated in the GM, whereas MYH2 was significantly upregulated in GM, consistent with their distinct contractile properties. In the GM, TNNT3 was significantly upregulated, indicating that this protein is a key factor in fast-twitch fiber formation [40]. Pyruvate kinase (PKM) and hexokinase-2 (HK2) are key enzymes in the glycolytic pathway and were significantly upregulated in GM, suggesting that fast-twitch muscle has a stronger glycolytic capacity than slow-twitch muscle [41]. However, this also implies relatively weaker lipid oxidation, which is unfavorable for the accumulation of lipid-derived flavor precursors. GO enrichment analysis further linked the functions of the DEPs to two major modules: lipid metabolism and energy metabolism. DEPs were significantly enriched in mitochondrial components (mitochondrial inner membrane, mitochondrial protein complexes) and biological processes related to fatty acid metabolism (fatty acid β-oxidation and lipid modification). This provides a structural basis for the enrichment of phospholipids, such as CL, PE, and PC, in the ST muscle, thereby preserving a richer pool of lipid substrates for flavor generation during thermal processing. In addition, the glycolytic enzymes (PKM, HK2) upregulated in GM complement the higher TG levels observed in the lipidomic profile of GM, suggesting that carbon sources in fast-twitch muscle are mainly directed towards glycogen breakdown and TG storage rather than phospholipid synthesis and accumulation [42]. KEGG pathway enrichment analysis anchored the functions of DEPs to three core metabolic pathways that determine the differences in flavor precursors. The most significantly enriched pathway was fatty acid metabolism, indicating that disparities in lipid metabolism are the most prominent molecular features distinguishing the two muscle types. Oxidative phosphorylation is the primary ATP supply pathway in slow-twitch fibers, and the predominant expression of related proteins in ST fibers is consistent with the high fatigue resistance of slow-twitch muscle [43,44]. This provides a protein-level explanation for the enrichment of CL and PE in ST: abundant mitochondria not only require CL to maintain their structural integrity but also depend on oxidative phosphorylation enzyme complexes to perform their functions, and together, these factors shape the flavor precursor lipid profile of ST muscle. In contrast, the enrichment of the glycolysis/gluconeogenesis pathway aligns with the physiological reliance of fast-twitch muscle on glycolysis for energy supply, but at the cost of a relatively diminished lipid oxidation capacity, which is consistent with the lower phospholipid levels and reduced flavor-forming potential observed in GM.

To elucidate how protein differences drive lipid changes, we integrated lipidomic and proteomic data. The slow-twitch (ST) muscle marker CA3 correlated positively with PE and PC, confirming ST phospholipid enrichment at the protein level [45], and negatively with SM, indicating membrane lipid differences between fiber types. KEGG enrichment showed that differential lipids and proteins co-mapped to glycerophospholipid metabolism and lipolysis regulation. The former aligns with high PE/PC and mitochondrial proteins in ST [46], explaining its larger flavor-precursor lipid pool; the latter suggests greater free fatty acid release during heating, directly supplying aroma precursors like aldehydes and alcohols [47]. Together, these findings support ST’s flavor advantage at the molecular level, with higher phospholipid content and oxidative metabolism providing the material basis for richer flavor compound generation during cooking.

In summary, by integrating lipidomics and proteomics, this study systematically elucidated the differential characteristics of fast- and slow-twitch muscles in Turpan black sheep at the levels of lipid molecules and protein expression. Oxidative ST is enriched with phospholipids (PE, PC, CL) and umami-related amino acids, conferring a greater potential for flavor development, whereas glycolytic GM is characterized primarily by triacylglycerols and sweet-tasting amino acids. The fundamental divergence in energy metabolism between these two muscle types is the key driving force behind the different profiles of flavor precursor substances. These findings provide new molecular insights into how muscle fiber type influences lamb flavor quality and offer a theoretical basis for improving lamb flavor through genetic selection and nutritional regulation.

Beyond statistical significance, the observed differences between slow-twitch (ST) and fast-twitch (GM) muscles may have practical implications for meat processing and marketing. If the TAV-based differences in umami and sweet amino acids exceed perceptible thresholds (as discussed above), these findings could inform product differentiation strategies. For example, ST cuts, which are rich in umami-related amino acids and phospholipids, might be preferentially positioned for cooking methods that maximize flavor development (e.g., roasting or grilling), whereas GM cuts, characterized by higher sweet amino acids and triacylglycerol contents, might be suited for different culinary applications or further processing. In markets where consumers place a premium on intense meat flavor and umami taste, the ST muscle might command a price premium. Conversely, in markets where milder or sweeter flavor profiles are preferred, the GM muscle may be equally or more acceptable. However, it is important to emphasize that consumer preferences are influenced by a complex interplay of flavor, texture, appearance, and cultural factors [48]. Therefore, the commercial relevance of the molecular differences reported here cannot be assumed and requires validation through consumer acceptance studies targeting specific market segments.

Several limitations of this study should be acknowledged. First, although our lipidomic and proteomic analyses have identified molecular differences between ST and GM muscles that may underlie flavor quality, this study did not include volatile compound analysis of cooked meat or sensory evaluation using trained taste panels. As emphasized throughout the discussion, statistically significant differences in precursor concentrations do not equate to consumer-perceptible differences in flavor. Without direct sensory data, any statements regarding flavor differences between the two muscles should be regarded as hypotheses or literature-based interpretations rather than definitive conclusions. The TAV-assessment presented here are intended to prioritize which molecular differences warrant further sensory investigation, not to replace sensory evaluation itself. Second, this study examined only two muscle locations from a single breed (Turpan black sheep). The generalizability of the identified candidate markers to other breeds, feeding systems, and rearing conditions remains to be verified. Third, the relationship between precursor concentrations and volatile compound formation during thermal processing is influenced by multiple variables, including heating parameters and the Maillard reaction, which were not examined in this study [49]. Future studies should: (1)conduct volatile flavor compound analysis on cooked ST and GM samples to establish the direct link between precursor lipids and aroma generation; (2) perform sensory evaluation using trained panels to determine whether the observed molecular differences translate into perceptible sensory differences; (3) conduct consumer acceptance studies to assess whether any sensory differences influence hedonic ratings and purchase intent in specific target markets; and (4) expand the sample scope to include multiple breeds and rearing conditions to validate the robustness of the identified markers.

## 5. Conclusions

In conclusion, our results demonstrate that the semitendinosus (ST) muscle is predominantly slow-twitch (oxidative) fiber-dominant, whereas the gluteus medius (GM) muscle is predominantly fast-twitch (glycolytic) fiber-dominant. By integrating lipidomics and proteomics, this study systematically characterized the molecular differences between these two muscle types and identified 389 differentially abundant lipids and 480 differentially expressed proteins. These differential molecules were mainly enriched in pathways related to the accumulation of flavor precursors, such as glycerophospholipid metabolism and lipolysis regulation. Joint analysis revealed a coordinated relationship between protein expression differences and changes in lipid composition, suggesting that protein differences are a key driver of lipid composition. In addition, PE, PC, CL, C20:1n-9, HK2, PKM, MYH7, and MYH2 were identified as key candidate markers. These findings require further validation in larger sample sizes and across more breeds. This study provides novel theoretical support for improving lamb flavor quality through genetic selection and nutritional regulation.

## Figures and Tables

**Table 1:** Amino acid contents of GM and ST muscles.

Amino Acid	Threshold (mg/g)	GM (mg/g)	GM SE	ST (mg/g)	ST SE	*p*-Value	TAV (GM)	TAV (ST)
Asp	1	8.41 ± 0.76 ^b^	0.44	10.12 ± 0.51 ^a^	0.29	0.032	8.41	10.12
Glu	0.3	19.31 ± 0.60 ^b^	0.34	22.62 ± 0.21 ^a^	0.12	0.001	64.35	75.39
Ser	1.5	4.88 ± 0.46 ^b^	0.26	6.65 ± 0.12 ^a^	0.07	0.016	3.25	4.43
Arg	2.6	13.66 ± 0.38 ^b^	0.22	19.03 ± 1.06 ^a^	0.61	0.001	5.25	7.32
Tyr	-	7.13 ± 0.81 ^b^	0.47	8.53 ± 0.15 ^a^	0.09	0.042	-	-
Val	0.4	7.88 ± 0.25 ^b^	0.15	9.95 ± 0.09 ^a^	0.05	0.002	19.70	24.88
Met	0.3	4.15 ± 0.16 ^b^	0.09	8.82 ± 0.42 ^a^	0.24	0.001	13.85	29.41
Ile	0.9	8.99 ± 0.20 ^b^	0.12	10.62 ± 0.13 ^a^	0.08	<0.001	9.99	11.80
Leu	1.9	12.78 ± 0.21 ^b^	0.12	13.68 ± 0.27 ^a^	0.16	0.011	6.72	7.20
His	0.2	5.75 ± 0.35 ^a^	0.20	4.21 ± 0.03 ^b^	0.02	0.016	28.75	21.07
Thr	0.6	6.55 ± 0.29 ^a^	0.17	5.91 ± 0.19 ^b^	0.11	0.034	10.91	9.85
Ala	0.5	15.82 ± 0.22 ^a^	0.13	10.53 ± 0.30 ^b^	0.18	<0.001	31.65	21.05
Pro	2.6	5.46 ± 0.51 ^a^	0.29	2.68 ± 0.30 ^b^	0.17	0.001	2.10	1.03
Gly	1.3	4.07 ± 0.69	0.40	4.50 ± 0.34	0.20	0.392	3.13	3.46
Phe	0.5	13.81 ± 1.15	0.66	15.23 ± 0.65	0.38	0.136	27.62	30.45
Lys	3	6.9 ± 1.05	0.61	9.22 ± 0.25	0.14	0.056	2.30	3.07
Cys	0.9	0.77 ± 0.08	0.05	0.71 ± 0.17	0.10	0.116	0.86	0.79
TAA	-	146.32 ± 1.84	1.06	163.01 ± 2.62	1.51	0.001	-	-

Different superscript letters (a, b) in the same row indicate significant differences between the two muscles (*p* < 0.05).

**Table 2:** Fatty acid contents of GM and ST muscles.

Fatty Acid	GM (mg/100 g)	GM SE	ST (mg/100 g)	ST SE	*p*-Value
C6:0	0.056 ± 0.009 ^b^	0.00	0.079 ± 0.01 ^a^	0.01	0.038
C8:0	0.224 ± 0.029	0.02	0.224 ± 0.01	0.01	0.978
C10:0	1.793 ± 0.296	0.17	1.883 ± 0.064	0.04	0.635
C11:0	0.014 ± 0.001 ^b^	0.00	0.018 ± 0.001 ^a^	0.00	<0.001
C12:0	0.698 ± 0.131	0.08	0.835 ± 0.042	0.02	0.157
C13:0	0.070 ± 0.011 ^b^	0.01	0.092 ± 0.004 ^a^	0.00	0.031
C14:0	18.768 ± 4.099	2.37	21.989 ± 0.672	0.39	0.250
C14:1n-5	0.405 ± 0.094	0.05	0.466 ± 0.034	0.02	0.347
C15:0	2.913 ± 0.699	0.40	3.855 ± 0.139	0.08	0.084
C16:0	329.909 ± 64.364	37.16	363.051 ± 10.067	5.81	0.428
C16:1n-7	16.835 ± 3.727	2.15	22.601 ± 1.365	0.79	0.066
C17:0	14.075 ± 3.538	2.04	17.455 ± 0.493	0.28	0.177
C18:0	246.728 ± 57.027	32.92	284.482 ± 7.561	4.37	0.319
C18:1n-9c	472.829 ± 89.915	51.91	571.028 ± 13.861	8.00	0.135
C18:2n-6c	94.009 ± 19.939	11.51	107.994 ± 3.159	1.82	0.296
C18:3n-6	1.333 ± 0.167	0.10	1.385 ± 0.059	0.03	0.639
C18:3n-3	6.359 ± 1.266	0.73	7.328 ± 0.243	0.14	0.263
C20:0	0.740 ± 0.196	0.11	1.065 ± 0.049	0.03	0.050
C20:1n-9	0.569 ± 0.131 ^b^	0.08	0.788 ± 0.025 ^a^	0.01	0.047
C20:2n-6	5.854 ± 1.265	0.73	6.100 ± 0.148	0.09	0.755
C20:3n-6	2.868 ± 0.461	0.27	2.955 ± 0.071	0.04	0.760
C20:4n-6	32.449 ± 7.218	4.17	36.56 ± 0.999	0.58	0.384
C20:5n-3	5.069 ± 0.980	0.57	4.413 ± 0.210	0.12	0.320
C22:0	0.158 ± 0.031	0.02	0.170 ± 0.015	0.01	0.573
C23:0	0.119 ± 0.003	0.00	0.117 ± 0.003	0.00	0.350
C24:0	0.401 ± 0.089	0.05	0.384 ± 0.01	0.01	0.756
C22:6n-3	2.486 ± 0.338	0.20	2.375 ± 0.076	0.04	0.608
SFA	616.667 ± 130.495	52.27	695.700 ± 19.088	7.79	0.358
MUFA	490.323 ± 93.862	38.32	594.883 ± 15.191	6.20	0.130
PUFA	150.427 ± 31.623	12.91	169.111 ± 4.835	1.97	0.369
PUFA/SFA	0.244 ± 0.001	-	0.243 ± 0.003	-	-

Different superscript letters (a, b) in the same row indicate significant differences between the two muscles (*p* < 0.05).

## Data Availability

The original contributions presented in the study are included in the article; further inquiries can be directed to the corresponding author.
